# Editorial: Enactivism and active inference in the therapeutic alliance

**DOI:** 10.3389/fpsyg.2022.1042698

**Published:** 2022-10-26

**Authors:** Patrice Duquette, Francesco Cerritelli, Jorge E. Esteves

**Affiliations:** ^1^Private Practitioner, Birmingham, MI, United States; ^2^Clinical-Based Human Research Department, Foundation COME Collaboration, Pescara, Italy; ^3^Malta ICOM Educational, Gzira, Malta; ^4^Research Department, University College of Osteopathy, London, United Kingdom

**Keywords:** active inference, free energy principle, enactivism, therapeutic alliance, biobehavioral synchrony, psychotherapy

The brain has no knowledge of reality but access only to the evidence of its sense organs. Thus, we can only know the world by actively inferring the causes of our sensations. The brain builds generative predictive models of the likely causes of sensory input from the individual's inner (interoceptive or proprioceptive) and outer (exteroceptive) environment. These models infer the most likely cause of incoming sensation, and this inference is a “prediction” of what will happen next. Predictions can be considered “beliefs,” but they are unconscious, subpersonal, and probabilistic inferences about what the cause of the sensation might be.

The Free Energy Principle provides an over-arching principle of how these processes support life. This holds that the embodied brain must minimize the difference between what the generative model predicts the sensation should be and what the sensation actually is, to lessen life-dispersing entropy, i.e., to minimize free energy. A discrepancy between what is predicted and what happens is termed “prediction error”—ostensibly uncertainty. Essentially, generative models are actively constructed, i.e., require action upon the sensory input. We actively sample data to build and maintain them through the process known as “active inference.” The process is also referred to as “enactive inference,” stressing the enactive nature of the embodied organism, interacting with its environment.

Processes of model building and updating may become inflexible or disturbed, for many reasons, with resulting pathology. Regardless of whether the pathology is expressed in the body or in the mind, aberrant belief updating is always involved (Friston, 2022).

The therapeutic alliance, involving dyadic and group exchanges, creates interpersonal synchrony that serves as a relational anchor to the patient's (en)active inference processes and facilitates increased flexibility in these inference processes. The papers in this special issue represent different disciplines and clinical approaches but are linked by how the therapeutic alliance can be employed, from the perspective of enactive inference, to promote effective treatment.

Hauke and Lohr posit that to alter the patient's habitual exploitation of limited model evidence, the therapeutic relationship must support exploratory strategies that introduce new data into model updating, from multiple sensory sources. Exploratory actions expand the sensory field and allow the development of alternative priors. In Cognitive Behavioral Therapy this includes mutually designing experiences that allow the patient to experiment with “directed exploration,” for example, exploring historical narratives within the treatment hour and exposing related physical and emotional reactions, followed by similar exercises. The strength of the therapeutic alliance can be reflected in the patient's commitment to goal-directed work.

Synchrony is a stabilizing aspect of the therapeutic alliance, as the treatment creates disorganization within the patient's habitual generative models. Synchrony is likened to “singing from the same hymn sheet” (Friston and Frith, 2015), as each individual agent's generative model moves closer to the other's, to minimize their own free energy within the relationship. Connolly explores this process within the psychotherapeutic dyad, describing how the individual generative models converge, under the influence of the therapist's model, which is held with greater precision/confidence. It is an outgrowth of mutual predictability that such entrainment occurs for both individuals. As Connolly explains, this is the juncture where the work of therapy can begin, as synchronized activity promotes “the emergence of a new hierarchical level in the client's generative model,” reducing the false precision/confidence of their previous beliefs.

Several papers consider the role of touch in enabling model updating, within an osteopathic framework of care. Kim et al. address distortions in interoceptive and proprioceptive inference that has led to faulty inference. Touch, coupled with purposeful verbal guidance of the patient's attention to sensory experience, and suggestions of new and different explanations for these sensations, allows the patient to reconsider old hypotheses. New sensations can be paired with new “rules” (i.e., predictions) about the cause of these sensations, thus establishing healthier generative models.

Touch, guided by verbal interactions, is similarly discussed by Bohlen et al., who propose that this increases the accurate perception of interoception information, with subsequent prediction error generation that updates faulty priors. An improvement in physiological dysfunction can result, for example, in better-regulated autonomic activity. They propose that osteopathic care, as an adjunct to psychotherapeutic approaches, can increase the chance of health-promoting shifts in the patient's model updating.

Esteves et al. consider how persistent physical pathology, e.g., chronic pain, can be treated with touch and directed verbal interaction by the clinician, which alters the patient's habitual attentional processes, develops synchrony, and alters the affordances that the environment offers to the individual. As new sensations develop, and the inference process is updated, these affordances allow the creation of a new shared narrative, elevated by “mutually predictive sensations” that benefit the patient's sense of self and relationships. A “shared ecological niche” is created, specific to the therapeutic dyad.

How touch generates biobehavioral synchrony and contributes to an alignment between clinician and patient, through mutual sensory experience, is explored by McParlin et al. “Ostensive cues” that are offered to the patient through touch, alongside the consistent and empathetic stance of the clinician, specifically lend predictability to the interaction, lessening uncertainty. This bidirectional aspect of touch furthers the therapeutic alliance, creating opportunities for generative model updating.

Importantly, exploration amidst model certainty generates destabilizing uncertainty, instigating a state of relative “chaos” (Connolly) in the patient's generative models as they explore new states. As these papers emphasize, the therapeutic alliance is the essential element that enables the patient to weather this uncertainty, without pushing against new model development, or regressing to old model exploitation. Whatever the discipline, the clinician can empathetically elicit the narrative that may reveal dysfunctional belief updating and support the change process with relational stability, as the patient is helped to foment instability that will update their previously strongly-held models and benefit them over a time span that they have no way to measure. Metaphorically, this can be viewed as the clinician listening carefully to the patient's verbal and physical expressions, initially offering a hymn sheet, and then persisting in helping them create their own.

## Author contributions

PD wrote the first draft of the manuscript. All authors contributed to the revision of the manuscript, approved, and accountable for the submitted manuscript.

## Conflict of interest

The authors declare that the research was conducted in the absence of any commercial or financial relationships that could be construed as a potential conflict of interest.

## Publisher's note

All claims expressed in this article are solely those of the authors and do not necessarily represent those of their affiliated organizations, or those of the publisher, the editors and the reviewers. Any product that may be evaluated in this article, or claim that may be made by its manufacturer, is not guaranteed or endorsed by the publisher.
